# Biomechanics of the ankle

**DOI:** 10.1016/j.mporth.2016.04.015

**Published:** 2016-06

**Authors:** Claire L. Brockett, Graham J. Chapman

**Affiliations:** **Claire L Brockett PhD** University Academic Fellow, Institute of Medical and Biological Engineering, University of Leeds, Leeds, UK. *Conflicts of interest: none declared*; **Graham J Chapman PhD** Research Fellow, Institute of Rheumatic and Musculoskeletal Medicine, University of Leeds and, Leeds NIHR Biomedical Research Unit, Leeds, UK. *Conflicts of interest: none declared*

**Keywords:** ankle biomechanics, subtalar joint, talocrural joint, tibiotalar joint

## Abstract

This paper provides an introduction to the biomechanics of the ankle, introducing the bony anatomy involved in motion of the foot and ankle. The complexity of the ankle anatomy has a significant influence on the biomechanical performance of the joint, and this paper discusses the motions of the ankle joint complex, and the joints at which it is proposed they occur. It provides insight into the ligaments that are critical to the stability and function of the ankle joint. It describes the movements involved in a normal gait cycle, and also highlights how these may change as a result of surgical intervention such as total joint replacement or fusion.

## Introduction

The ankle joint complex is comprised of the lower leg and the foot and forms the kinetic linkage allowing the lower limb to interact with the ground, a key requirement for gait and other activities of daily living. Despite bearing high compressive and shear forces during gait, the ankle's bony and ligamentous structure enables it to function with a high degree of stability, and compared with other joints such as the hip or knee, it appears far less susceptible to degenerative processes such as osteoarthritis, unless associated with prior trauma. This paper will highlight key anatomical bony structures and soft tissues that form the ankle joint complex and will further highlight how the ankle joint complex functions during walking and how pathology changes these movements.

## Anatomy of the ankle

The foot and ankle is made up of the twenty-six individual bones of the foot, together with the long-bones of the lower limb to form a total of thirty-three joints.^1^ Although frequently referred to as the ‘ankle joint’, there are a number of articulations which facilitate motion of the foot. The ankle joint complex is made up of the talocalcaneal (subtalar), tibiotalar (talocrural) and transverse-tarsal (talocalcaneonavicular) joint.

### The subtalar joint

The calcaneus is the largest, strongest and most posterior bone of the foot, providing attachment for the Achilles tendon. It is located inferiorly to the talus, and forms a triplanar, uniaxial joint with the talus.^2^ The talus rests on the anterior portion of the calcaneus. The two similarly articulated facets of the anterior talocalcaneal joint on the inferior aspect of the talus are convex, and on the superior aspect of the calcaneus are concave, while the facets for articulation of the posterior talocalcaneal joint on the inferior aspect of the talus are concave, and on the superior aspect of the calcaneus are convex. This geometry allows inversion and eversion of the ankle, and whilst other motion is permitted at this joint, most of eversion and inversion of the foot is provided here.^3^ A number of ligaments form attachments between the two bony surfaces. The key linkage between the two is the interosseous talocalcaneal ligament, a strong, thick ligament that extends from the articular facets of the inferior talus to the superior surface of the calcaneus. Two further ligaments, the lateral talocalcaneal ligament and the anterior talocalcaneal ligament also contribute to the connection of this joint,^1^ however these are relatively weak. The talocalcaneal joint is also supported by the calcaneofibular part of the lateral collateral ligament and the tibiocalcaneal ligament of the deltoid. Furthermore, the long tendons of peroneus longus, peroneus brevis, flexor hallucis longus, tibialis posterior, and flexor digitorum longus provide additional support.^4^

### The tibiotalar joint (Talocrural joint)

The tibiotalar joint forms the junction between the distal tibia and fibula of the lower leg and the talus. The load-bearing aspect of this joint is the tibial-talar interface. The talus bone includes the head, neck and body, and has no direct muscle connection. The trochlea of the talus fits into the mortise formed from the distal ends of the long bones of the shin. The malleoli of the tibia and fibula act to constrain the talus, such that the joint functions as a hinge joint, and primarily contributes to the plantar- and dorsiflexion motion of the foot. However, the geometry of the joint, such as the cone-shaped trochlea surface and the oblique rotation axis do indicate it may not function simply as a hinge.^1^, ^4^ The talus is at its widest anteriorly, meaning the joint is more stable in dorsiflexion.^5^ The conforming geometry of the tibiotalar joint is considered to contribute to the stability of the joint. In stance phase, the geometry of the joint alone is sufficient to provide resistance to eversion; otherwise stability is derived from the soft tissue structures.

**Figure 1:**
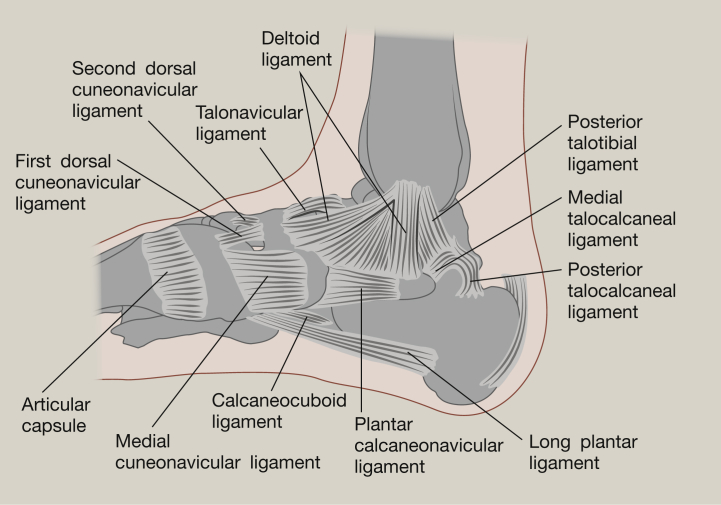
Medial ligaments of the tibiotalar joint.

**Figure 2:**
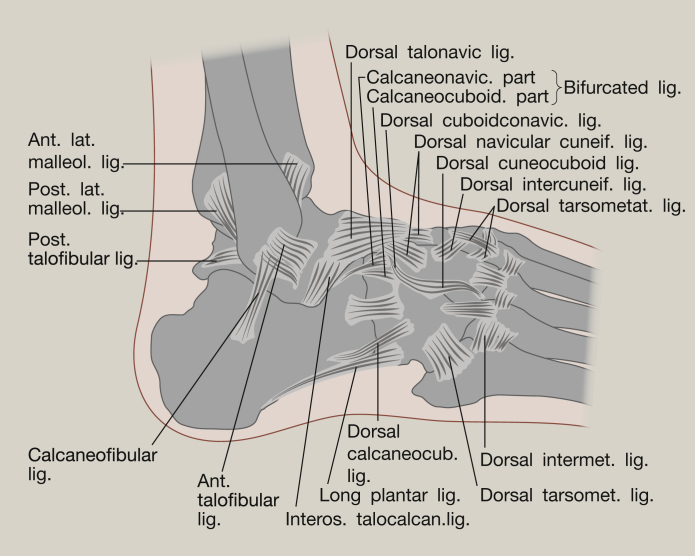
Lateral ligaments of the ankle.

The tibiotalar joint is a diarthrosis and is covered by a thin capsule attaching superiorly to the tibia, and the malleoli, and inferiorly to the talus. Stability is given to the joint through three groups of ligaments. The tibiofibular syndesmosis limits motion between the tibia and fibula during activities of daily living, maintaining stability between the bone ends. The syndesmosis consists of three parts – the anterior tibiofibular ligament, the posterior tibiofibular ligament and the interosseous tibiofibular joint.^1^, ^5^ The medial aspect of this ankle joint is supported by the medial collateral ligaments (or deltoid ligaments) and these are key to resisting eversion motion and valgus stresses within the joint^1^ (Figure 1). The deltoid ligament is fan shaped and comprises the anterior and posterior tibiotalar ligaments, the tibionavicular ligament and the tibiocalcaneal ligament. The lateral collateral ligaments reduce inversion of the joint, limiting varus stresses and reduce rotation. They consist of the anterior and posterior talofibular ligaments and the calcaneofibular ligament (Figure 2). The anterior and posterior ligaments withstand high tensile forces under plantar and dorsiflexion respectively. These ligaments provide stability to the lateral tibiotalar joint,^4^, ^5^, ^6^ and are frequently damaged during inversion injuries such as ankle sprain. The calcaneofibular ligament is the only direct connective tissue between the tibiotalar and subtalar joints.

### Inferior tibiofibular joint

This joint has already been referred to in the explanation of the tibiotalar joint. In some literature it is considered as a core aspect of the tibiotalar joint, but may also be considered as a distinct joint.^7^ This is not a synovial articulating joint, the interosseous membrane, a fibrous tissue, connects the two distal portions of the fibula and tibia.^6^ The primary function of this joint is a stabilizing role, adding stability, rather than additional motion to the foot and ankle. As previously detailed, the anterior and posterior tibiofibular ligaments and interosseous ligament maintain the joint between the tibia and fibula. The ligamentous constraint of the joint makes it highly susceptible to injury, and is often involved in ankle fracture and eversion injuries.

### Transverse tarsal joint

The transverse tarsal joint (Chopart's joint) combines the junction between the talus and navicular, where anteriorly, the talar head articulates with the posterior aspect of the navicular, and the calcaneocuboid joint, the joint between the calcaneus and the cuboid. The transverse tarsal joint is considered as part of the same functional unit as the subtalar joint as they share a common axis of motion,^3^, ^4^ also contributing to eversion-inversion motion of the foot.

### Muscles of the ankle

The majority of motion within the foot and ankle is produced by the twelve extrinsic muscles, which originate within the leg and insert within the foot. These muscles are contained within four compartments. The anterior compartment consists of four muscles: the tibialis anterior, the extensor digitorum longus, the extensor hallucis longus, and the peroneus tertius. The tibialis anterior and the extensor hallucis longus produce dorsiflexion and inversion of the foot. The peroneus tertius produces dorsiflexion and eversion of the foot. The extensor digitorum longus only produces dorsiflexion of the foot. The lateral compartment is composed of two muscles: the peroneus longus and the peroneus brevis, which produce plantarflexion and eversion of the foot. The posterior compartment consists of three muscles: the gastrocnemius, the soleus, and the plantaris, which contribute to plantarflexion of the foot. The deep posterior compartment is composed of three muscles: the tibialis posterior, the flexor digitorum longus, and the flexor hallucis longus, which produce plantarflexion and inversion of the foot.^1^, ^6^

## Biomechanics of the ankle

### Motion of the foot and ankle

**Figure 3:**
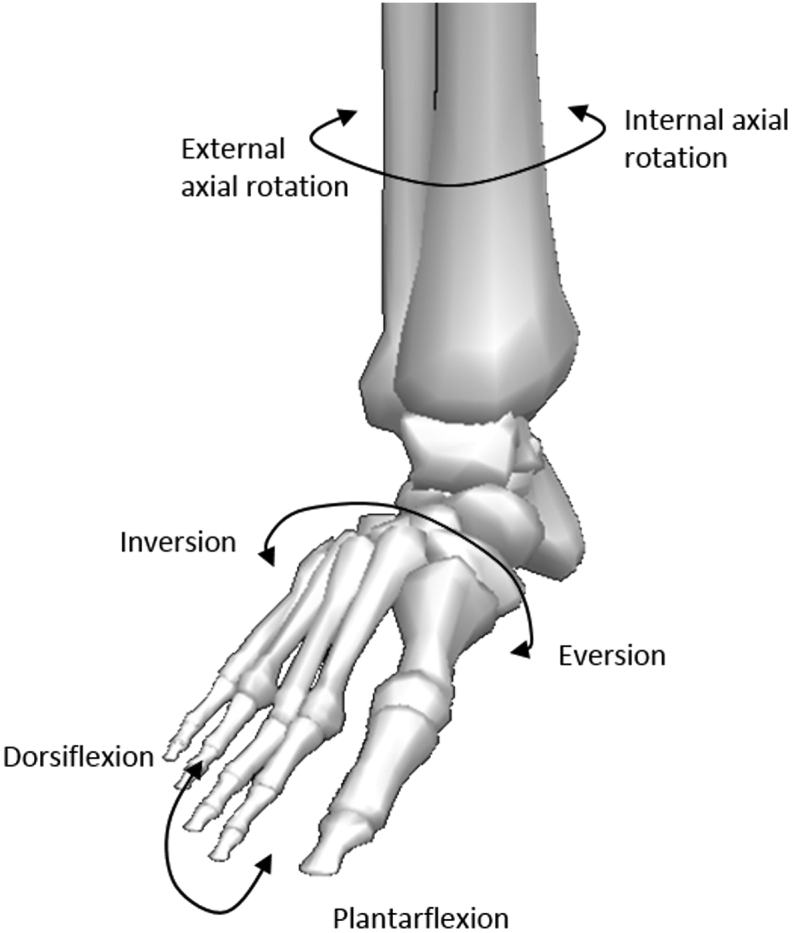
Diagram illustrating relative motions of the ankle joint complex. Figure adapted from Visual 3D (C-Motion, Rockville, Maryland).

The key movement of the ankle joint complex are plantar- and dorsiflexion, occurring in the sagittal plane; ab-/adduction occurring in the transverse plane and inversion-eversion, occurring in the frontal plane^8^ (Figure 3). Combinations of these motions across both the subtalar and tibiotalar joints create three-dimensional motions called supination and pronation.^5^ Both terms define the position of the plantar surface of the foot (sole). During supination, a combination of plantarflexion, inversion and adduction causes the sole to face medially. In pronation, dorsiflexion, eversion and abduction act to position the sole facing laterally.

### Axis of rotation of the ankle

**Figure 4:**
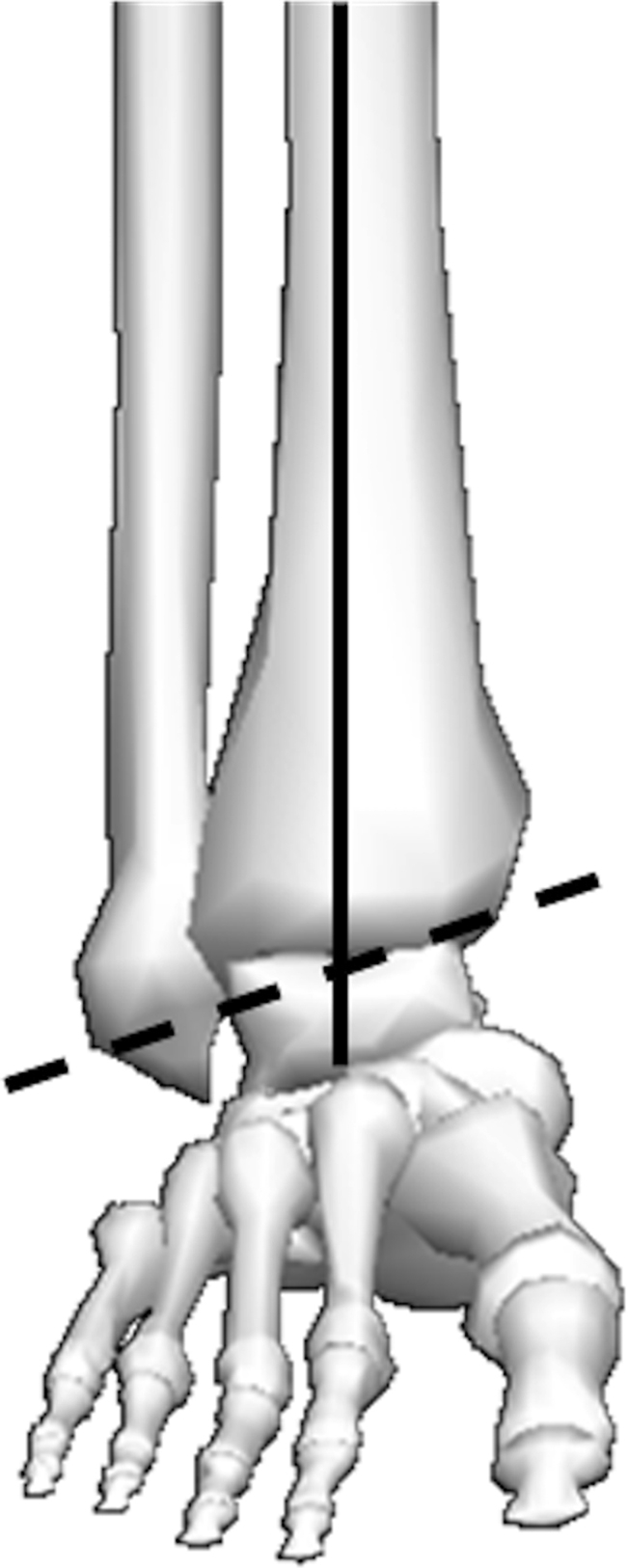
Diagram illustrating the sagittal and frontal plane axis of rotation for the ankle joint complex. Dashed line represents the axis of rotation for the dorsiflexion and plantarflexion. The intersecting point between the bold and dashed line represents the point of rotation for inversion and eversion. Figure adapted from Visual 3D (C-Motion, Rockville, Maryland).

**Figure 5:**
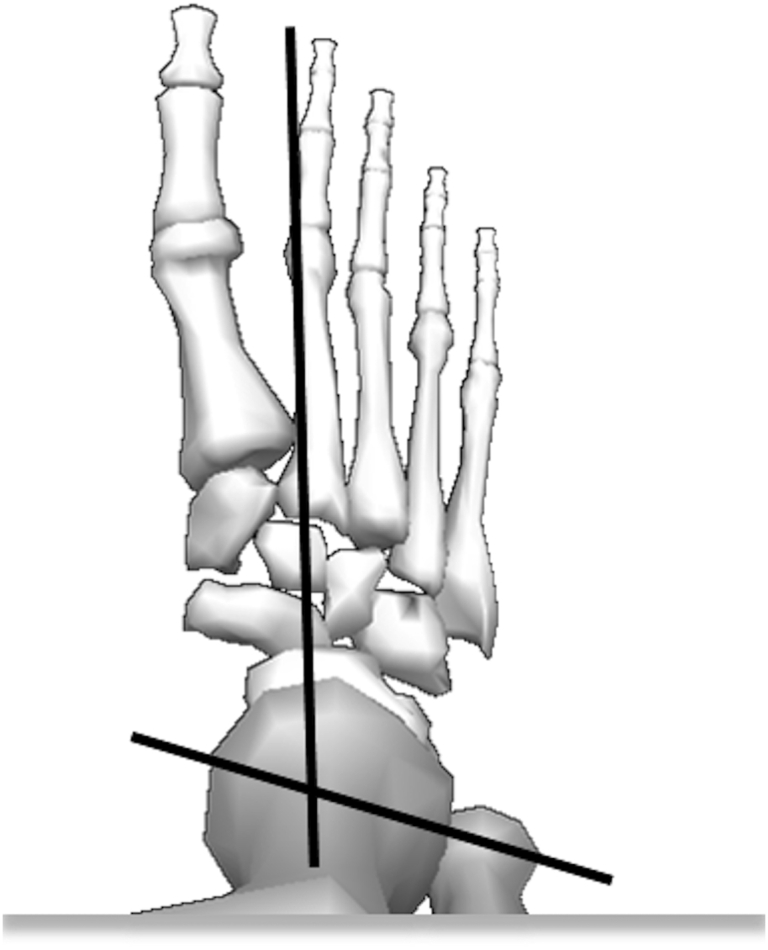
Diagram illustrating the ankle joint complex axis of rotation in the transverse plane. The intersecting point represents the point of rotation for internal and external foot progression (toe in or toe out gait). Figure adapted from Visual 3D (C-Motion, Rockville, Maryland).

Whilst many authors consider the tibiotalar joint to be a simple hinge joint, there has been some suggestion that it is multi-axial, due to the internal rotation that occurs during dorsiflexion, and the external rotation that occurs in plantarflexion. However, there is evidence to suggest the tibiotalar joint is indeed uniaxial, but the simultaneous motion observed occur as a result of its oblique axis.^4^, ^9^ The axis of rotation of the ankle joint complex in the sagittal plane occurs around the line passing through the medial and lateral malleoli (dotted line, Figure 4). The coronal plane axis of rotation occurs around the intersecting point between the malleoli and the long axis of the tibia in the frontal plane (Figure 4). The transverse plane axis of rotation occurs around the long axis of the tibia intersecting the midline of foot (Figure 5).

Studies of the talar anatomy have highlighted the difference in radial curvature in the medial and lateral aspects, indicating the axis of rotation of the ankle joint will vary as motion changes.^10^ Based on this, a number of authors have proposed multiple axes of motion for the ankle joint during normal activity. Since the 1950s,^9^, ^10^ it has been proposed there are a plantarflexion axis, which points upwards towards the lateral side of the ankle joint, and a dorsiflexion axis which is inclined downwards and laterally (Figure 4). These are parallel in the transverse plane, but can differ by up to 30° in the coronal plane. Motion about these axes cannot occur simultaneously, and the transition between axes during motion is estimated to occur close to the neutral position of the joint.^11^

The axis of the subtalar joint is also an oblique axis, running from posterior to anterior forming an angle of approximately 40° with the anteroposterior axis in the sagittal plane, and forming an angle of 23° with midline of foot in the transverse plane. In a similar way to the tibiotalar joint, the subtalar joint creates multiple motion during plantar and dorsiflexion, resulting in pronation and supination.^11^

### Range of motion

The ankle range of motion (ROM) has been shown to vary significantly between individuals due to geographical and cultural differences based on their activities of daily living,^12^ in addition to the method used for assessing ROM. Motion of the ankle occurs primarily in the sagittal plane, with plantar- and dorsiflexion occurring predominantly at the tibiotalar joint. Several studies have indicated an overall ROM in the sagittal plane of between 65 and 75°, moving from 10 to 20° of dorsiflexion through to 40–55° of plantarflexion.^12^, ^13^ The total range of motion in the frontal plane is approximately 35° (23° inversion − 12° eversion).^13^ However, in everyday activities, the ROM required in the sagittal plane is much reduced, with a maximum of 30° for walking, and 37° and 56° for ascending and descending stairs, respectively.^5^ Historically there has been a convention where dorsi- and plantarflexion motion was solely attributed to the tibiotalar joint motion, and inversion–eversion was considered to occur only at the subtalar joint. More recently, the complete separation of the motions to each joint has been dismissed; most plantar/dorsiflexion is still considered to occur at the tibiotalar joint but with a few degrees accounted for at the subtalar joint.^14^ The distribution of inversion and/or eversion and rotation across the two joints has been an area of greater contention, with some studies indicating eversion to occur at the subtalar joint and rotation/inversion to occur at the tibiotalar, whereas others have shown version to be distributed across both joints.^15^ Whilst gait analysis can be used as an objective tool for quantifying motion of lower limb joints and forces that act upon these joints, gait analysis cannot separate the talocalcaneal (subtalar), tibiotalar (talocrural) and transverse-tarsal (talocalcaneonavicular) joint due to the major limitation of accurately measuring talus motion using skin-mounted markers. However, despite this limitation, gait analysis is still a commonly used tool for the quantification of ankle joint complex kinematics and kinetics.

**Figure 6:**
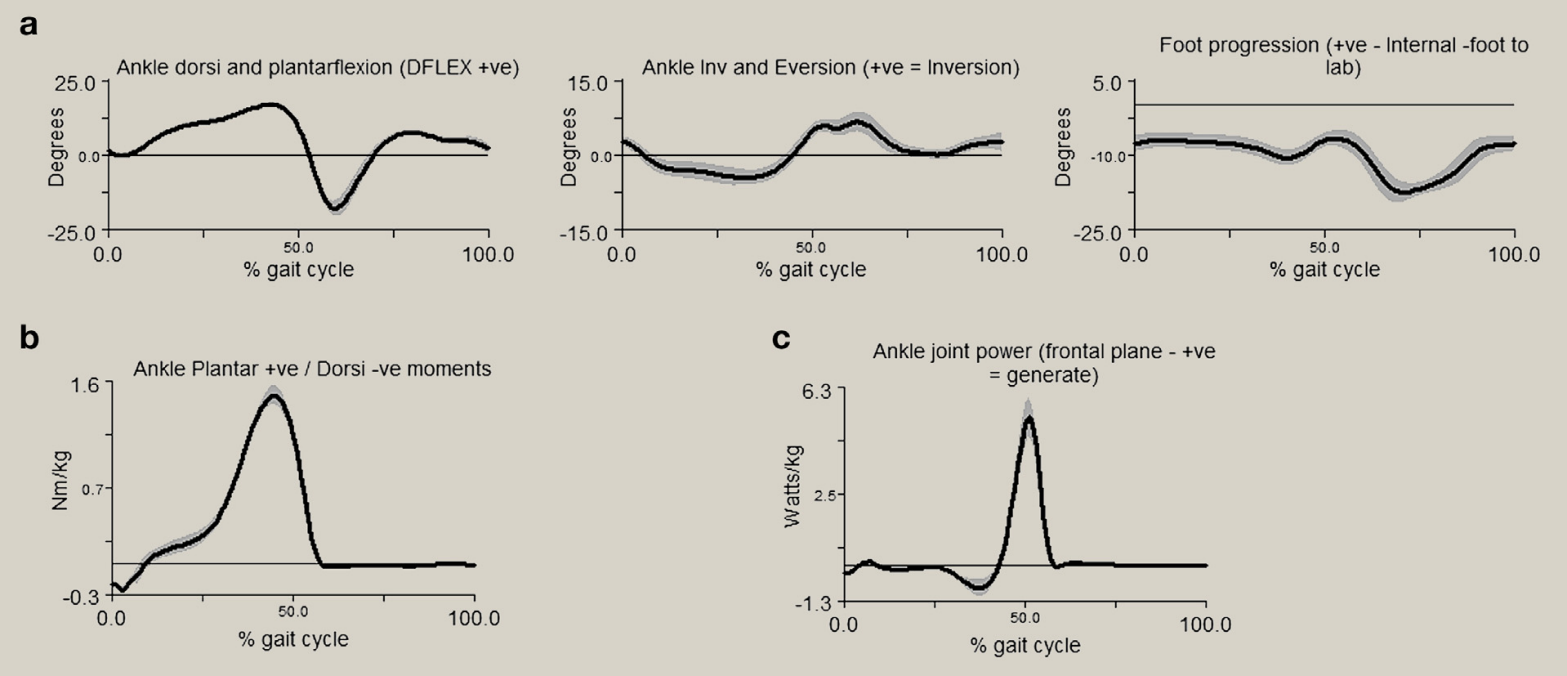
Diagram illustrating typical outputs from gait analysis of five walking trials. a) representing ankle complex rotation in sagittal, frontal and transverse planes (left to right, respectively); b) sagittal plane ankle moments and c) sagittal plane ankle power. The shaded area on all graphs represents ±1 standard deviation. Figure adapted from Visual 3D (C-Motion, Rockville, Maryland).

Figure 6 depicts example gait analysis data of the ankle joint complex kinematics, kinetics and powers. During a normal gait cycle, the stance phase can be split into three sub-phases based on the sagittal motion of the ankle; i) the heel rocker; ii) the ankle rocker and iii) the forefoot rocker. The heel rocker phase begins at heel strike, where the ankle is in a slight plantarflexed position pivoting around the calcaneus (the continuation of plantarflexion) until the end of the heel rocker phase when the foot is flat on the ground. During this sub-phase the dorsiflexors are eccentrically contracting to lower the foot to the ground. The ankle rocker phase is where the ankle moves from plantarflexion to dorsiflexion during which the shank (tibia and fibula) rotate forward around the ankle allowing forward progression of the body. During the forefoot rocker phase, the foot rotates around the forefoot phase, starting when the calcaneus lifts off the ground evident by the ankle beginning to plantarflex and continuing until maximum plantarflexion (approximately 14°) being achieved at toe-off, where power generation is achieved for the leg to begin the swing phase. During swing phase the ankle dorsiflexes enabling the foot to clear the ground and avoiding stumbling/tripping, before returning to slight plantarflexion at heel strike. This flexion motion is complemented by motion at the sub-talar joint, with approximately 15° of eversion/inversion. For the majority of individuals, inversion occurs at heel-strike, and progresses to eversion during mid-stance phase, allowing the heel to rise and push off into swing.^5^

### Forces in the ankle joint

The ankle joint complex bears a force of approximately five times body weight during stance in normal walking, and up to thirteen times body weight during activities such as running.^16^ The ankle moment obtained from gait analysis (see Figure 6b) demonstrates a dorsiflexion moment at heel strike as the dorsiflexors eccentrically contract to control the rotation of the foot onto the ground and prevent the foot from slapping the ground. During the second phase, there is a plantarflexor moment as the ankle dorsiflexors contract eccentrically to allow forward progression of the shank over the foot. During the third phase, the plantar flexion moment continues with the plantar flexors contracting concentrically towards toe-off. As walking speed increases, ankle kinetic patterns remain similar in profile but with greater magnitudes. Ankle joint moments acquired from gait analysis do not commonly report ankle moments in the coronal or transverse planes due to the complex nature of movement of the ankle joint complex and the high variability between individuals.

Ankle power (Figure 6c) varies when the major muscles acting on the ankle joint complex are either absorbing or generating power during gait. The negative values correspond with power absorption from the plantar flexors eccentrically contracting during the heel and ankle rocker phases. The maximum joint power of the ankle joint complex is generated at approximately 50% of gait cycle during the forefoot rocker phase corresponding with the power generation of the plantarflexors required for the lower limb to propel the body forward towards toe-off.

Experimental studies have indicated that approximately 83% of load is transmitted through the tibiotalar joint, with the remaining 17% transmitted through the fibula.^15^ The amount of load transferred through the fibula varies, with increased loading occurring during dorsiflexion. Of the load carried across the tibial-talar joint, between 77% and 90% is applied to the talar dome, with the remaining load distributed across the medial and lateral surfaces.^3^ This load distribution is a function of both ligamentous forces and positional effects, with the medial facet experiencing highest load during inversion, and the lateral facet exposed to highest load during eversion.

The ankle has a relatively high level of congruency, meaning that despite experiencing high loads during normal activities, the load-bearing area of the ankle is large (11–13 cm^2^), and it has been proposed that this should result in lower stress than at the hip or knee.^5^ The majority of contact analysis within the ankle has been conducted through computational prediction or cadaveric experimentation, which clearly have limitations for assessing *in-vivo* conditions. A statically applied load of 1.5 kN (approximately twice body weight) in a cadaveric study, with the ankle in a neutral position demonstrated a mean contact pressure of 9.9 MPa, and a contact area of 483 mm^2^, significantly less than the area proposed previously.^17^ Exploration of the pressures under static loading with the ankle in positions reflecting phases of the gait cycle indicate that contact pressures are generally higher in plantarflexion than in dorsiflexion.^15^ Weight bearing MRI and fluoroscopy has shown that the largest contact area occurs during the stance phase of gait, with lower contact at both toe-off and heel strike.^18^

## Clinical relevance of ankle biomechanics

Age and gender are both influential factors that may change ankle ROM. A study compared gender differences within different age groups, between 20 and 80 year of age.^19^ This demonstrated that younger females (20–39 years old) have a higher ankle ROM compared to males. However, with increasing age, older females demonstrated 8° less dorsiflexion and 8° greater plantar flexion compared to male patients in the oldest age group (70–79 years old). Additionally, there was a reduction in ROM for both genders in the oldest age groups.

Degenerative processes of the foot and ankle, such as post-traumatic osteoarthritis may have a significant impact on the biomechanical function of the ankle. Compared to the hip and knee, post-traumatic osteoarthritis is more prevalent. There have been a number of studies undertaken to explore the impact of ankle surgery on ankle biomechanics. Common surgical interventions for end-stage ankle OA include total ankle replacement or ankle arthrodesis, both, aimed at improving pain and function of the patient. Joint replacement has been transformative for hip and knee osteoarthritis but for total ankle replacement, problems remain. Gait analysis can be used as a useful objective tool for measuring functional performance of patients following a surgical intervention. Patients with end-stage ankle osteoarthritis typically walk more slowly, have a reduced ankle ROM and have altered ankle moments compared to healthy controls.^20^, ^21^ Following a total ankle replacement, the literature describes an improvement in walking speed, various spatio-temporal measures, ankle ROM and sagittal plane ankle moments.^20^, ^21^, ^22^, ^23^, ^24^, ^25^ Despite these improvements which indicate improved functional performance, a number of gait parameters remain diminished. For example, ankle joint moments and power remain significantly reduced.^24^, ^25^ This suggests the forces acting on/around the ankle are potential limiting factors for improvements in post-operative function compared to that of the natural ankle.

Ankle arthrodesis represents a functionally more conservative alternative with less risk of future requirement for revision. Fusion of the joint, by its nature, limits the function of the tibio-talar joint, and in some cases subtalar fusion can be undertaken simultaneously, effectively locking the ankle in a fixed position. Gait analysis performed pre- and post-arthrodesis surgery has also demonstrated improvements in walking speeds and spatio-temporal measures.^22^, ^23^, ^26^, ^27^ However post-operative gait parameters are still significantly diminished. The reduced motion often results in hypermobility of the midfoot causing adjacent joint OA.^28^, ^29^ Other complications following ankle arthrodesis include pain, dysfunction, non-union and malalignment.^28^, ^29^, ^30^ Neither ankle replacement or ankle arthrodesis restores ankle normal function however total ankle replacement would appear to provide greater post-operative improvements when compared with ankle arthrodesis.

## Summary

The anatomy of the ankle joint complex determines that the biomechanics is not just that of a simple hinge joint but that of multi-axial motions occurring simultaneously to facilitate human gait. Simple factors such as gender and age can impact on the biomechanics of the ankle, and diseases such as arthritis can influence the range of motion and ankle power. Surgical treatment for end stage degeneration significantly influences the biomechanical function of the ankle, and has a notable impact on the surrounding joints.

## Figures and Tables

By Henry Vandyke Carter – Henry Gray (1918) Anatomy of the Human Body (See “Book” section below) Bartleby.com: Gray's Anatomy, Plate 354, Public Domain, https://commons.wikimedia.org/w/index.php?curid=537826).

(By Henry Vandyke Carter – Henry Gray (1918) Anatomy of the Human Body (See “Book” section below) Bartleby.com: Gray's Anatomy, Plate 355, Public Domain, https://commons.wikimedia.org/wiki/File%3AGray355.png).
